# Correction: Meier et al. Hantavirus Replication Cycle—An Updated Structural Virology Perspective. *Viruses* 2021, *13*, 1561

**DOI:** 10.3390/v15020273

**Published:** 2023-01-18

**Authors:** Kristina Meier, Sigurdur R. Thorkelsson, Emmanuelle R. J. Quemin, Maria Rosenthal

**Affiliations:** 1Department of Virology, Bernhard Nocht Institute for Tropical Medicine, 20359 Hamburg, Germany; 2Centre for Structural Systems Biology, Leibniz Institute for Experimental Virology, University of Hamburg, 22607 Hamburg, Germany; 3Fraunhofer Institute for Translational Medicine and Pharmacology ITMP, 22525 Hamburg, Germany

## Text Correction

There was an error in the original publication [1] in the following sentences: “Interestingly, the cytoplasmic tail of Gc (comprising 110 residues) has a size that is very similar to that of the Z protein specific to arenaviruses [128], which is not expressed by members of other bunyavirus families. Similar to the zinc-binding Z protein, the hantavirus Gc cytoplasmic tail was shown to contain a zinc-coordination site [113], and both have the same localization within the virion, lining the inner side of the viral envelope [128].” It should be Gn instead of Gc in both sentences.

## References

In addition, there is an error in the reference for the following sentence: “The dissociation of the Gn/Gc complexes at a low pH is thought to make the tripartite fusion loop accessible for membrane fusion, although this dissociation does not seem to trigger membrane fusion and seems to even be reversible [39].” The reference here in the original publication is “Hepojoki, J.; Strandin, T.; Wang, H.; Vapalahti, O.; Vaheri, A.; Lankinen, H. Cytoplasmic tails of hantavirus glycoproteins interact with the nucleocapsid protein. *J. Gen. Virol.*
**2010**, *91*, 2341–2350.” (It has been changed to Ref. [112] in the updated version). The correct reference is “Rissanen, I.; Stass, R.; Zeltina, A.; Li, S.; Hepojoki, J.; Harlos, K.; Gilbert, R.J.C.; Huiskonen, J.T.; Bowden, T.A.; Sundquist, W.I. Structural Transitions of the Conserved and Metastable Hantaviral Glycoprotein Envelope. *J. Virol.*
**2017**, *91*.” (Original Ref. [40]). With this correction, the order of some references has been adjusted accordingly.

The authors state that the scientific conclusions are unaffected. This correction was approved by the Academic Editor. The original publication has also been updated.
